# *Amphidinium* spp. as a Source of Antimicrobial, Antifungal, and Anticancer Compounds

**DOI:** 10.3390/life13112164

**Published:** 2023-11-04

**Authors:** Ida Orefice, Sergio Balzano, Giovanna Romano, Angela Sardo

**Affiliations:** Department of Ecosustainable Marine Biotechnology, Stazione Zoologica Anton Dohrn, Via Ammiraglio Ferdinando Acton 55, 80131 Naples, Italy; ida.orefice@szn.it (I.O.); sergio.balzano@szn.it (S.B.); giovanna.romano@szn.it (G.R.)

**Keywords:** *Amphidinium*, dinoflagellates, biological activity, anticancer, antifungal, antimicrobial compounds

## Abstract

Dinoflagellates make up the second largest marine group of marine unicellular eukaryotes in the world ocean and comprise both heterotrophic and autotrophic species, encompassing a wide genetic and chemical diversity. They produce a plethora of secondary metabolites that can be toxic to other species and are mainly used against predators and competing species. Dinoflagellates are indeed often responsible for harmful algal bloom, where their toxic secondary metabolites can accumulate along the food chain, leading to significant damages to the ecosystem and human health. Secondary metabolites from dinoflagellates have been widely investigated for potential biomedical applications and have revealed multiple antimicrobial, antifungal, and anticancer properties. Species from the genus *Amphidinium* seem to be particularly interesting for the production of medically relevant compounds. The present review aims at summarising current knowledge on the diversity and the pharmaceutical properties of secondary metabolites from the genus *Amphidinium*. Specifically, *Amphidinium* spp. produce a range of polyketides possessing cytotoxic activities such as amphidinolides, caribenolides, amphidinins, and amphidinols. Potent antimicrobial properties against antibiotic-resistant bacterial strains have been observed for several amphidinins. Amphidinols revealed instead strong activities against infectious fungi such as *Candida albicans* and *Aspergillus fumigatus*. Finally, compounds such as amphidinolides, isocaribenolide-I, and chlorohydrin 2 revealed potent cytotoxic activities against different cancer cell lines. Overall, the wide variety of antimicrobial, antifungal, and anticancer properties of secondary metabolites from *Amphidinium* spp. make this genus a highly suitable candidate for future medical applications, spanning from cancer drugs to antimicrobial products that are alternatives to currently available antibiotic and antimycotic products.

## 1. Introduction

**Figure 1:**
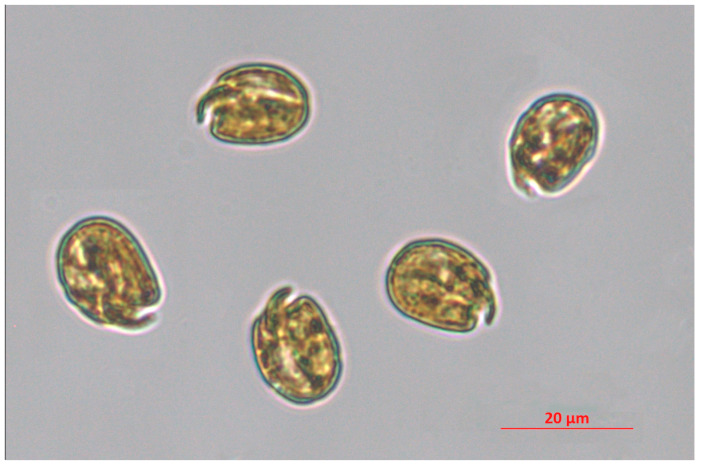
*Amphidinium carterae* CCMP 1314. This species is included in the culture collection of the Ecosustainable Marine Biotechnology Department at SZN, and the micrograph was taken through an inverted microscope Zeiss Axio Observer 7 (Jena, Germany) at 200× magnitude by I. Orefice.

Dinoflagellates are a group of microalgae widely distributed in freshwater and marine environments, which comprise autotrophic, heterotrophic, and mixotrophic species. A number of dinoflagellate species have been described as symbionts (e.g., *Symbiodinium*, *Pelagodinium*), parasites (e.g., *Amoebophrya, Ichthyodinium*), and grazers (e.g., *Gyrodinium*) [1,2,3,4,5,6,7]. The number of species belonging to this taxon has been recently estimated at ca. 6000 species; among them, more than 60% are living and the remaining part represent fossil species [8]. The variety of feeding behaviours is comparable to their biochemical diversity, that leads to the production of a plethora of secondary metabolites, most of them possessing significant biological activities towards cancer cell lines, bacteria, viruses, fungi, larvae, and other algae [9,10,11,12,13,14,15,16]. Among them, some toxic molecules (saxitoxin, tetrodotoxin, okadaic acid) have been largely investigated to assess their potential in the pharmaceutical field since the 2000s [17]. However, toxins can be detrimental for human health, especially when massively released in the water column during harmful algal blooms (HABs). HAB frequency has been constantly increasing over the last few decades because of climate change and coastal eutrophication [18,19,20]. Among the huge species richness of dinoflagellates, the genus *Amphidinium* seems to be particularly relevant for its high potential in producing bioactive metabolites. Species belonging to this genus, along with other closely related genera, produce several poliketides, including amphidinols, amphidinolids, amphidinins, and iriomoteolides, and lots of these secondary metabolites possess significant cytotoxic activity, which is described in the present article. With respect to other dinoflagellates, usually characterised by low growth rates [21], *Amphidinium* spp. are able to perform rapid growth, and reach high abundances and relatively high biomass yields under appropriate culturing conditions [22,23]. This could be advantageous in a perspective of a large-scale production of metabolites potentially marketable in the industrial sector. Moreover, modulation of culture conditions, such as light intensity and nutrient supply, can further promote the production of specific metabolites [24]. In the present paper, we collected information available in literature about the most significant biological activities of *Amphidinium* spp.-derived metabolites, highlighting the potential of this genus as a source of bioactive compounds including pharmaceuticals, but also the main bottlenecks currently avoiding the commercialisation of their bioactive metabolites. Figure 1 represents cells of the strain *Amphidinium carterae* CCMP 1314 (also known as FE102 clone).

Antimicrobial resistance developed by some pathogens is a serious public health issue that needs to be resolved through the common efforts of the scientific community, society, and policy makers. An increasing number of infectious diseases caused by different pathogens, such as bacteria, parasites, viruses, and fungi, are difficult to prevent and to treat because of adaptation mechanisms evolved by several distinct pathogens to overcome the action of several commonly used drugs. Antimicrobial resistance has been reported to cause ca. 700,000 fatalities per year worldwide [25]. In particular, bacterial resistance to antibiotics seems to be the microbe-driven drug adaptation strategy leading to the most serious issues for human health; indeed, several bacterial species exhibit antibiotic resistance, and bacterial infections can often lead to severe consequences [26]. Bacteria exhibiting the most dangerous drug adaptation patterns are multidrug-resistant strains affiliated to the species *Staphylococcus aureus*, *Escherichia coli*, *Enterococcus faecium*, *Streptococcus pneumoniae*, *Klebsiella pneumoniae*, and *Pseudomonas aeruginosa* [27]. Innovative and high-quality antibacterial compounds are thus urgently required to replace the antibiotics that are going to be rendered increasingly ineffective by drug resistance [28]. Since most of the antibiotics known to date have been developed from natural products, the marine environment can represent a promising source, still little explored, of new bioactive compounds with antibacterial activity. Although the dinoflagellate *Amphidinium* produces a plethora of secondary metabolites with numerous bioactive properties, few studies have investigated the potential of *Amphidinium* spp. as a source of antibacterial molecules. The first evidence was reported by Kubota and co-authors [29], in which the amphidinolide Q, a cytotoxic 12-membered macrolide, and four new 4,5-seco-analogues, namely amphidinins C, D, E, and F, were identified in the liquid medium in which *Amphidinium* sp. strain 2012-7-4A was cultured. This strain was isolated from the marine flatworm *Amphiscolops* sp. collected at Ishigaki, Okinawa, Japan. The antibacterial activity was evaluated against two Gram-positive bacteria, *S. aureus* and *Bacillus subtilis*, and a Gram-negative bacterium, *E. coli*. Results demonstrated that amphidinins C and E and amphidinolide Q were active against *S. aureus* and *B. subtilis*, while only amphidinolide Q was effective against *E. coli* (minimum inhibitory concentration—MIC—of 32 µg/mL for all trials except *B. subtilis* treated with amphidinolide Q, MIC of 16 µg/mL (Table 1)). Amphidinins D and F and the glycosides-related compounds did not show antibacterial activity. More recently, Barone and co-authors [30] evaluated the antibacterial activity of *Amphidinium carterae* strain LACW11, isolated on the west Irish coast, against two Gram-positive bacteria, *S. aureus* and *E. faecalis*. The activity was detected mainly in three fractions obtained by ethyl acetate extraction and C_18_ fractionation with increasing percentages of methanol, namely fractions J (80% methanol), I (90% methanol), and K (100% methanol), with an MIC ranging from 16 µg/mL to 64 µg/mL for *S. aureus* and from 64 µg/mL to 256 µg/mL for *E. faecalis* (Table 1). The chemical identification of these fractions, through a metabolomic approach, highlighted the presence of amphidinol AM-A and a new derivative, dehydroAM-A, in fractions I and J, respectively. These two compounds were mostly responsible for the antibacterial activity against *S. aureus*. Fraction K, which showed bioactivity against *E. faecalis*, did not contain known amphidinols, suggesting the presence of other bioactive molecules in this fraction. Antimicrobial activity occurs throughout nature; there are many examples of bioactive secondary metabolites produced by a variety of both land-based and underwater sources [31]. Terrestrial and marine secondary metabolites have different structural features and bioactive proprieties, probably due to the different environmental characteristics in which the original organisms occur [32]. Chassagne and co-authors [33] reported a systematic analysis of scientific data about plants possessing significant antibacterial activities, selecting data on 958 species derived from 483 scientific articles. This analysis indicated the crude extracts of the plant species *Sambucus nigra* L. (Adoxaceae), *Echinops kebericho* Mesfin (Asteraceae), *Mikania glomerata* Spreng. (Asteraceae), *Curcuma longa* L. (Zingiberaceae), and *Combretum album* Pers., (Combretaceae) as those with the most potent antibacterial activity, with MIC values ranging from 3.5–16 μg/mL, comparable or slightly lower compared to MIC of *Amphidinium*-related compounds. Essential oils are concentrated hydrophobic liquids extracted from plants; they are generally very complex in terms of chemical composition, showing a powerful antibacterial activity, with MIC values that reached 0.09 μg/mL for the plant *Hibiscus surattensis* L. (Malvaceae), probably due to the synergistic effect between the different compounds present in the extracts. In addition to antibacterial activities, the species *A. carterae* has been also investigated for its antialgal and antilarval activity, as reported by Kong and co-authors [9]. These properties were tested to find environmentally friendly antifouling compounds for marine industries. A series of unsaturated and saturated 16- to 22-carbon fatty acids, including hexadecanoic acid, octadecanoic acid, 9-octadecenoic acid, octadecatetraenoic acid, eicosapentaenoic acid (EPA), and docosahexaenoic acid, exhibited antialgal activity against the diatom *Skeletonema costatum*, as indicated by changes in the chlorophyll *a* fluorescence intensity of the microalgal suspension, and antilarval activity against *Amphibalanus amphitrite* larvae with relatively low lethal concentrations. The antimicrobial properties of fatty acids isolated from marine organisms are well documented [34]. The type and potency of bioactivity depends on the chemical structure, in terms of degree of saturation, length of carbon chain, and the orientation of the double bonds [35]. Among the most promising fatty acids, EPA showed potent activity against different bacteria [36], and palmitic acid revealed antialgal activity and antifouling properties against the diatom *Cylindrotheca closterium* with a half-maximal effective concentration (EC_50_) value of 45.5 μg/mL [37]. However, literature data on the most used antifouling agents, such as diuron, copper thiocyanate, and tolylfluanid, showed a more powerful antialgal activity, with an EC_50_ always lower than 1 μg/mL, compared to compounds isolated from natural sources [38,39,40]. On the other hand, the use of chemical agents represents a serious environmental risk, due to their persistence and toxicity to nontarget organisms. Recently, the stricter restrictions on the European Community have limited the use of these chemical agents, leading to a growing need to find alternatives to synthetic antifouling compounds [41]; within this context, further efforts to prove the validity of *Amphidinium* spp. as a valuable natural source of antifouling molecules are mandatory.

## 2. Antifungal Activity

Fungal infections represent a serious clinical problem, especially for immunocompromised and seriously ill patients. Among clinical infections, those caused by the species *Aspergillus niger* and *Candida albicans* can cause morbidity and mortality when associated with other diseases, advanced age, and/or patients who have undergone an organ transplant [42]. Dinoflagellates include a high proportion (ca. 70%) of species possessing biocide activity against fungal infections [43], and most of them were tested on the abovementioned fungi [12,44,45]. Some potent dinoflagellate-derived compounds with antifungal activity were isolated over 30 years ago from the species *Gamberdiscus toxicus* [46], identified as polyether compounds termed as gambieric acids [47]. However, most of the antifungal compounds derive from the genus *Amphidinium*. In less recent works (from the late 1990s to the first decade of the 2000s), the biological activity of *Amphidinium* compounds was evaluated by susceptibility tests based on paper disks impregnated of specific concentrations of the agent, which were aimed at identifying the minimum effective concentration (MEC) able to inhibit fungal proliferation [48,49]. More precise in vitro assays were performed to detect the MIC of the antifungal agents during the last decade. MIC can be assessed by colorimetric assays based on the reduction of resazurin, a nonfluorescent blue dye that is reduced to the pink-coloured resorufin [50], or by the broth microdilution method [51], which is based on the inoculation of a standardised number of organisms in a liquid medium exposed to serial dilutions of an antifungal agent [12]. MIC values of *Amphidinium*-derived compounds (Table 2) are comparable or slightly higher than organic extracts obtained from natural compounds of plant origin, such as *Abutilon theophrasti*, *Acacia nilotica*, *Cinnamomum verum* and *Ficus polita*, while they are—as expected—lower than plant-derived essential oils [52]. Amphidinols can be also considered equally or more effective than other antifungal compounds isolated from some marine organisms [53], such as the bacterium *Acinetobacter* sp., which produces indolepyrazines exhibiting a MIC of 12–14 µg/mL against *C. albicans* [54], and the fungus *Penicillum* sp., which contains andrastone C and andrastone B exhibiting MIC values against *C. albicans* of 6 and 13 μg/mL, respectively [55]. The antifungal effects exhibited by compounds from *Amphidinium* spp. and other marine organisms towards aspergillosis is comparable (MIC values have the same order of magnitude) to plant-derived solvent extracts from the families Asteraceae and Lamiaceae, but generally produce a more marked effect of flavonoids and phenolic compounds extracted from other several species (families: Fabaceae, Aizoaceae, Anacardiaceae, Hypericaceae, Cornaceae, Bignoniaceae, Aquifoliaceae) that possess MIC values of 0.01–6.25 mg/mL [56]. Antifungal activity of Amphidinol A, C, and 18 against *C. albicans* can be considered also comparable to that of fluconazole, one of the most common synthetic compounds that is actually considered as one of the mainstays for the treatment of *Candida*-derived infections. Indeed, MIC values of these compounds are inside or below the range of the susceptible dose-dependent (SDD, MIC: 16–32 µg/mL) clinical breakpoint for fluconazole and *Candida*, and below the resistant (R; MIC ≥ 64 µg/mL) breakpoint [57]. Few data regarding *Amphidinium*-derived molecules with antifungal activity are available in the literature with respect to those of synthetic compounds (e.g., triazoles), and the application of different methodologies can make the direct comparison among these natural compounds with the most common antifungal agents difficult. Moreover, the increasing resistance exhibited by *Candida* and *Aspergillus* species towards the most common biocides [58,59,60] have already highlighted the need to test and validate the efficiency of novel azoles with improved spectra of activity [61]; we hypothesise that this research can be extended also to natural sources, including marine bioactive compounds from *Amphidinium* spp.

## 3. Anticancer Activity

In addition to antimicrobial and antifungal activities, the genus *Amphidinium* has been widely investigated for the anticancer properties of its secondary metabolites. Both symbiotic and free-living *Amphidinium* spp. have been reported to possess several anticancer compounds. An extensive review on the anticancer properties of macrolides and polyketides produced by *Amphidinium* spp. is provided by Kobayashi and Tsuda [65]. Overall, these authors classified all the amphidinolides isolated and identified back in 2006, and reported the presence of 34 cytotoxic amphidinolides in 7 *Amphidinium* sp. strains. The first bioactive compounds were isolated from an *Amphidinium* sp. symbiont of the flatworm *Amphiscolops breviviridis* and named amphidinolides B, G, and H [66]. In particular, amphidinolides G and H have proven to be very effective against murine leukemia cells (Table 3). Amphidinolide B, Amphidinolide H, and amphidinolide H3 revealed instead cytotoxic activity against murine leukemia and human epidermoid carcinoma (Table 3). Amphidinol-22 was isolated from crude extracts of *A. carterae* and was found to exhibit moderate cytotoxic activity against lung, liver, and pancreas cancer cell lines [62]. The amphidinolides most effective against cancer cells are Amphidinolide N, which was found to exhibit potent cytotoxic activity against human cervix adenocarcinoma cells—half maximal inhibitory concentration (IC_50_) = 0.01 ng/mL—and Amphidinolides B and H, which were revealed to be effective against murine leukemia (IC_50_ = 0.14 and 0.48 ng/mL, respectively, Table 3). In addition to amphidinolides, *Amphidinium* spp. possess other long-chain compounds such as luteophanols, colopsinols, and caribenolide, the latter possessing cytotoxic properties [67]. The most potent anticancer compounds identified to date are two macrolides (isocaribenolide-I and chlorohydrin-2) recently isolated from a free-living *Amphidinium* sp. (strain KCA09053) and found to possess high cytotoxic activity against human cervix adenocarcinoma cells [68]. Caribenolide I, isolated from a free-living *Amphidinium* sp., was found to possess cytotoxic activities towards human colon tumour cells [69]. Tsuda et al. [70] identified amphidinolide U while Kobayashi et al. [71] isolated amphidinolides T2, T3, and T4, from a symbiotic *Amphidinium* sp.. These amphidinolides exhibit moderate cytotoxic activity (IC_50_ = 7–12 μg/mL) against murine leukemia (L1210) [70,71]. Since the concentration of amphidinolides can be, in most cases, extremely low within *Amphidinium* spp. cells, several authors synthesised amphidinolides in the laboratory; for example, Lu et al. [72] synthesised amphidinolide B2 and demonstrated its activity against human solid (IC_50_ = 1.6 ng/mL) and blood tumour cells (53.9 ng/mL). Furstner et al. [73] published a protocol for the synthesis of the most effective amphidinolides: Amphidinolides B, D, G, and H. In addition, very potent anticancer molecules (IC_50_ = 68 ng/mL against melanoma cell lines A2058) similar to amphidinols have been recently isolated from the Octocoral *Stragulum bicolor* [74]. Overall, on the one hand laboratory synthesis of amphidinolides allows for the selection of specific compounds and the implementation of a single synthetic process, leading to amphidinolides at a higher degree of purity, on the other hand mass culturing *Amphidinium* spp. under the appropriate physical and chemical conditions may lead to the production and the extraction of these amphidinolides without applying complex laboratory protocols. The comparison between these two alternative processes for the production of selected anticancer compounds needs to be evaluated for each specific molecule. 

## 4. Discussion

**Figure 2:**
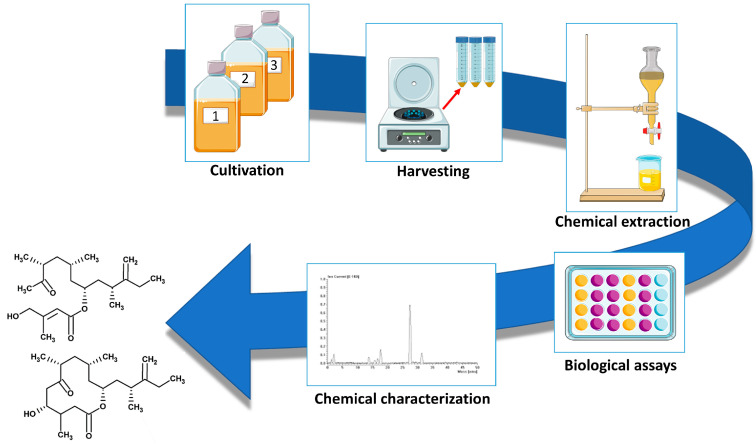
Pipeline for isolation and characterisation of novel bioactive compounds from the genus *Amphidinium*. Images downloaded from https://smart.servier.com/ (accessed on 2 October 2023).

The great variety of biological activities associated with natural products (NPs) renders them an attractive source for drug discovery. They represent, indeed, ca. one-third of the new molecular entities approved from the Food and Drug Administration [85]. With respect to synthetic compounds, NPs show a greater structural diversity which results in a higher spectrum of biological functionalities [86]. However, in a perspective of a large-scale production of NP-derived drugs, the main bottleneck of the use of natural sources is related to their massive consumption. For example, huge amounts of arable land and clean water are required for the exploitation of plant-derived NPs, posing serious risks of competition in the use of arable land between food production and NPs production. The use of aquatic animals for the massive production of NPs carries instead the risk of incidental release in the environment, which, in case of allochthonous species, can seriously alter ecosystem health. Among NPs, ca. 28,500 compounds have been identified from marine sources [87], most of them exhibiting cytotoxic and anticancer properties [88], and a high proportion of marine natural products (MNPs) derives from microorganisms [87], including microalgae [89,90]. The true distinctive strength of exploiting microalgae as a source of natural compounds is the possibility of culturing them in areas that do not enter competition for land and space with other organisms. Moreover, microalgae show high growth rates with respect to the natural life cycle of other organisms and are not strictly influenced by seasonal variations, especially if cultured in enclosed systems. To date, the massive production of microalgae is mostly limited to few species belonging to the genera *Dunaliella*, *Chlorella*, *Haematococcus, Tisochrysis, Tetraselmis*, and *Schizochytrium*, which are usually employed as live feed in aquaculture or as source of food and feed ingredients [91,92]. Among dinoflagellates, the only species that has been cultured at an industrial scale to produce docosahexaenoic acid as a dietary supplement was *Crypthecodinium cohnii* [92]. Recently, some efforts were made in using pilot-scale systems as predictive models for large scale-cultivation of the genus *Amphidinium* [23,93], to produce high-valuable compounds such as polyunsaturated fatty acids [93] and also to define strategies to increase the production of amphidinols [24]. *Amphidinium* spp. can attain high cell densities during the stationary phase [44] and are widely distributed in almost all temperate and warm marine environments, as evidenced by the high number of the isolates currently available in the culture collections (https://ncma.bigelow.org/search?keywords=amphidinium&page=1, accessed on 2 October 2023; https://www.ccap.ac.uk/catalogue/index.php?route=product/search&search=amphidinium&mfp=61-archived; accessed on 2 October 2023), in which the collection site is indicated. Their ubiquity, the great variety of secondary metabolites possessing a plethora of biological activities, and the relatively high growth rate that ensures a sufficient amount of biological material for the study of bioactive compounds, make the genus *Amphidinium* an attractive source of natural-derived antifungal, antimicrobial, and anticancer compounds. The pipeline to detect bioactive compounds in the genus *Amphidinium* in shown in Figure 2; this process is similar for most microalgae and other organisms. 

Current data suggest that light-driven oxidative stress and cultivation in nutrient-replete media can lead to amphidinol accumulation [24]. In addition, the biological activity from some amphidinols is extremely high and little amounts of biomass might be sufficient for a reliable extraction process. The high variability of secondary metabolites produced by this genus suggests that any novel strain could have a particular biological activity, that can be due to known or undiscovered compounds. However, deeper analyses of biological activities are to be carried out also on strains which have been already investigated, especially when specific biological activities of the total extract are higher than those of the purified compounds, suggesting the presence of other unidentified and active compounds [74]. To date, ca. 45 chemically characterised metabolites have shown a clear biological activity toward microbes, fungi, and/or specific cancer cell lines, but biological activities were also found in solvent or aqueous extracts and fatty acids of some species [9,30,69,84]. In addition, active compounds from *Amphidinium* spp. such as amphidinol 20, luteophanol D, and lingushuiol A are regularly excreted by the cells and can be recovered from the supernatant [94]; this means that aside from the optimisation of culture conditions and biomass harvesting, technical solutions to concentrate and recover bioactive compounds from the culture media should be assessed. Tuning culture conditions for a further improvement of growth rates and yields of *Amphidinium*-derived secondary metabolites is crucial to promote their industrial commercialisation, and, currently, available literature is still limited. Moreover, the production of polyketides for pharmaceutical purposes requires a very high degree of purity of dinoflagellate cultures, imposing further limits to large-scale production that rarely ensure sterile conditions. Aside from technical issues for the large-scale cultivation of these dinoflagellates, the real effectiveness of certain *Amphidinium*-derived compounds could be better elucidated. Indeed, some activities have been identified by using methods that do not allow for a precise quantification of the activity threshold [30,45,48], and the study of antifungal and antibacterial activities is mostly based on in vitro studies. Moreover, the duration of clinical trials should not be underestimated, in case of the approval of some compounds as potential drugs. In summary, there are still several limits that currently hamper the use of *Amphidinium*-derived metabolites in the pharmaceutical field, including (i) the diversity and distribution of such metabolites within different species, and their variability under different culturing conditions; (ii) contamination risks on large-scale cultivations; (iii) the scarce knowledge of their effects tested in vivo; (iv) the long-term processes once their efficacy has been validated. However, the increasing interest in NPs as alternative sources of synthetic, unsafe products, and the high pharmaceutical potential of the genus *Amphidinium*, are two valid reasons for further investigations aimed at enabling the commercialisation of novel, potential drugs.

## 5. Conclusions

The present paper describes the huge variety of metabolites isolated in the last 30 years by microalgal species belonging to the genus *Amphidinium*, and highlights the potential of these dinoflagellates as a source of pharmaceuticals. *Amphidinium* spp. have been shown to produce a diverse pool of specialty products possessing antimicrobial, antifungal, and anticancer properties. Although the long-term process of clinical trials is quite daunting, the great pharmaceutical potential of *Amphidinium* compounds with proven biomedical properties further supports the need for additional efforts in implementing microalgal mass culturing to produce these compounds on larger scale, in order to improve yields and decrease costs and operation times of industrial microalgal cultivation.

## Figures and Tables

**Table 1:** Major active compounds from *Amphidinium* spp. possessing antimicrobial properties. Abbreviations: MIC, minimum inhibitory concentration; EC_50_, half-maximal effective concentration, LC_50_, half-maximal lethal concentration. ^1^ Metanol/ethyl acetate extraction and SPE C18 fractionation (Fraction K with 100% methanol). ^2^ Mix of hexadecanoic acid, octadecanoic acid, 9-octadecenoic acid, octadecatetraenoic acid, eicosapentaenoic acid, and docosahexaenoic acid.

Compound or Extract	Type	Species	Properties	Target Microbs	Activity	Reference
Amphidinin C	Polyketide	*Amphidinium* sp. (2012-7-4A strain)	Antibacterial	*S. aureus*	MIC: 32 µg/mL	[29]
Amphidinin C	Polyketide	*Amphidinium* sp. (2012-7-4A strain)	Antibacterial	*B. subtilis*	MIC: 32 µg/mL	[29]
Amphidinin E	Polyketide	*Amphidinium* sp. (2012-7-4A strain)	Antibacterial	*S. aureus*	MIC: 32 µg/mL	[29]
Amphidinin E	Polyketide	*Amphidinium* sp. (2012-7-4A strain)	Antibacterial	*B. subtilis*	MIC: 32 µg/mL	[29]
Amphidinolide Q	Polyketide	*Amphidinium* sp. (2012-7-4A strain)	Antibacterial	*E. coli*	MIC: 32 µg/mL	[29]
Amphidinolide Q	Polyketide	*Amphidinium* sp. (2012-7-4A strain)	Antibacterial	*S. aureus*	MIC: 32 µg/mL	[29]
Amphidinolide Q	Polyketide	*Amphidinium* sp. (2012-7-4A strain)	Antibacterial	*B. subtilis*	MIC: 16 µg/mL	[29]
Amphidinol AM-A	Polyketide	*A. carterae* (LACW11 strain)	Antibacterial	*S. aureus*	MIC: 16 µg/mL	[30]
Amphidinol AM-A	Polyketide	*A. carterae* (LACW11 strain)	Antibacterial	*E. faecium*	MIC: 64 µg/mL	[30]
Amphidinol dehydroAM-A	Polyketide	*A. carterae* (LACW11 strain)	Antibacterial	*S. aureus*	MIC: 16 µg/mL	[30]
Amphidinol dehydroAM-A	Polyketide	*A. carterae* (LACW11 strain)	Antibacterial	*E. faecium*	MIC: 128 µg/mL	[30]
Methanol extract ^1^	NA	*A. carterae* (LACW11 strain)	Antibacterial	*S. aureus*	MIC: 64 µg/mL	[30]
Methanol extract ^1^	NA	*A. carterae* (LACW11 strain)	Antibacterial	*E. faecium*	MIC: 256 µg/mL	[30]
C_16–22_ fatty acids ^2^	Fatty acids	*A. carterae*	Antialgal	*S. costatum*	EC_50_ at 72 h: 12.9 μg/mL	[9]
C_16–22_ fatty acids ^2^	Fatty acids	*A. carterae*	Antilarval	*A. amphitrite*	LC50 at 24 h: 15.1 μg/mL	[9]

**Table 2:** Main antifungal agents isolated from *Amphidinium* spp. and related activity towards *Candida albicans* or *Aspergillus* species.

Compound	Species	Target Fungus	Activity	Reference
Amphidinol 22	*Amphidinium carterae*	*Aspergillus fumigatus*	MIC: 64 µg/mL; 100% growth inhibition at 560 µg/mL	[62]
Amphidinol 21	*Amphidinium carterae*	*Aspergillus niger*	MEC: <15 μg/disk	[49]
Amphidinol 20	*Amphidinium carterae*	*Aspergillus niger*	MEC: <15 μg/disk	[49]
Amphidinols 18	*Amphidinium carterae*	*Candida albicans*	MIC: 9 µg/mL	[12]
Amphidinol 13	*Amphidinium carterae*	*Aspergillus niger*	growth inhibition: 132.0 mg/disk	[45]
Amphidinol 12	*Amphidinium carterae*	*Aspergillus niger*	growth inhibition: >100 mg/disk	[45]
Amphidinol 11	*Amphidinium carterae*	*Aspergillus niger*	growth inhibition: 256.6 mg/disk	[45]
Amphidinol 10	*Amphidinium carterae*	*Aspergillus niger*	growth inhibition: 154.0 mg/disk	[45]
Amphidinol 9	*Amphidinium carterae*	*Aspergillus niger*	growth inhibition: 32.9 mg/disk	[45]
Amphidinol 7	*Amphidinium klebsii*	*Aspergillus niger*	MEC: 10 mg/disk	[48]
Amphidinol 4	*Amphidinium carterae*	*Aspergillus niger*	growth inhibition: 58.2 mg/disk	[45]
Amphidinol 2,6	*Amphidinium klebsii*	*Aspergillus niger*	growth inhibition: 6 µg/disk	[63]
Amphidinol 2	*Amphidinium carterae*	*Aspergillus niger*	growth inhibition: 44.3 mg/disk	[45]
Amphidinol C	*Amphidinium carterae*	*Aspergillus fumigatus, Candida albicans*	minimum fungicidal concentration: 8 µg/mL (*A. fumigatus*); 16 µg/mL (*C. albicans*)	[63,64]
Amphidinol A	*Amphidinium carterae*	*Candida albicans*	MIC: 19 µg/mL	[44,45]

**Table 3:** Main anticancer compounds/solvent extracts from *Amphidinium* spp. and related biological activity towards cancer cell lines.

Compound or Extract	Species	Target Cancer Cells	IC50	Reference
Isocaribenolide-I	*Amphidinium* sp. (strain KCA09053)	Human cervix adenocarcinoma cells	0.02 ng/mL	[68]
Chlorohydrin 2	*Amphidinium* sp. (strain KCA09053)	Human cervix adenocarcinoma cells	0.06 ng/mL	[68]
Amphidinolide N	*Amphidinium* sp. (strain KCA09053)	Human cervix adenocarcinoma cells	0.01 ng/mL	[68]
Amphidinolide B	*Amphidinium* sp.	Murine leukemia (L1210)	0.14 ng/mL	[67]
Amphidinolide H	*Amphidinium* sp.	Murine leukemia (L1210)	0.48 ng/mL	[66]
Amphidinolide H	*Amphidinium* sp.	Human epidermoid carcinoma	0.52 ng/mL	[66]
Amphidinolide N (Caribenolide I)	*Amphidinium operculatum*	Human colon cell lines	1 ng/mL	[69]
Amphidinolide H3	*Amphidinium* sp.	Murine leukemia (L1210)	2 ng/mL	[67]
Amphidinolide B	*Amphidinium* sp.	Human epidermoid carcinoma (KB)	4.2 ng/mL	[67]
Amphidinolide G	*Amphidinium* sp.	Murine leukemia (L1210)	5.4 ng/mL	[66]
Amphidinolide G	*Amphidinium* sp.	Human epidermoid carcinoma	5.9 ng/mL	[66]
Amphidinolide H3	*Amphidinium* sp.	Human epidermoid carcinoma (KB)	22 ng/mL	[67]
Iriomoteolide-3a	*Amphidinium* sp. (strain HYA024)	Human B lymphocyte DG-75	80 ng/mL	[75]
Lingshuiol	*Amphidinium* sp.	Human lung carcinoma (A549)	0.28 μg/mL	[76]
Lingshuiol	*Amphidinium* sp.	Promyelotic leukemia (HL-60)	0.31 μg/mL	[76]
90% aqueous methanol extract	*Amphidinium operculatum*	Human colon cell lines	0.35 μg/mL	[69]
Amphirionin-2	*Amphidinium* sp. (strain KCA09051)	Human colon carcinoma Caco-2	0.1 μg/mL	[77]
Iriomoteolide-13a	*Amphidinium* sp. (strain KCA09053)	Human cervix adenocarcinoma HeLa cells	0.5 μg/mL	[78]
Amphirionin-2	*Amphidinium* sp. (strain KCA09051)	Human lung adenocarcinoma A549	0.6 μg/mL	[77]
Iriomoteolides-11a	*Amphidinium* sp. (strain KCA09052)	Human cervix adenocarcinoma (HeLa)	0.7 μg/mL	[79]
Iriomoteolide-10a	*Amphidinium* sp. (strain KCA09053)	Human cervix adenocarcinoma (HeLa)	0.7 μg/mL	[80]
Iriomoteolide-4a	*Amphidinium* sp. (strain HYA024)	Human B lymphocyte DG-75	0.8 μg/mL	[81]
Iriomoteolide-5a	*Amphidinium* sp. (strain HYA024)	Human B lymphocyte DG-75	1.0 μg/mL	[81]
Iriomoteolide-10a	*Amphidinium* sp. (strain KCA09053)	Human B lymphocyte DG-75	0.9 μg/mL	[80]
Iriomoteolide-10a	*Amphidinium* sp. (strain KCA09053)	Murine hepatocellular carcinoma MH134	1.9 μg/mL	[80]
Amphidinolide V	*Amphidinium* sp.	Murine leukemia (L1210)	3.2 µg/mL	[82]
Amphidinolide V	*Amphidinium* sp.	Epidermoid carcinoma (KB)	7 μg/mL	[82]
Iriomoteolide-15a	*Amphidinium* sp. (strain KCA09052)	Human cervix adenocarcinoma (HeLa)	4 μg/mL	[83]
Iriomoteolide-14a	*Amphidinium* sp. (strain KCA09052)	Human cervix adenocarcinoma (HeLa)	4 μg/mL	[83]
Iriomoteolides-9a	*Amphidinium* sp. (strain KCA09052)	Human cervix adenocarcinoma (HeLa)	5.6 μg/mL	[79]
Amphidinolide T3	*Amphidinium* sp. (strain Y71)	Murine leukemia (L1210)	7 μg/mL	[71]
Amphidinolide T2	*Amphidinium* sp. (strain Y71)	Murine leukemia (L1210)	10 μg/mL	[71]
Amphidinolide T4	*Amphidinium* sp. (strain Y71)	Murine leukemia (L1210)	11 μg/mL	[71]
Amphidinolide U	*Amphidinium* sp.	Murine leukemia (L1210)	12 μg/mL	[70]
Amphidinol 22	*Amphidinium carterae*	Hepatocyte carcinoma HepG2	11 μg/mL	[62]
Amphidinol 22	*Amphidinium carterae*	Human lung carcinoma (A549)	13 μg/mL	[62]
Amphidinol 22	*Amphidinium carterae*	Pancreas carcinoma (MiaPaca)	14.9 μg/mL	[62]
Amphidinolide T1	*Amphidinium* sp. (strain Y71)	Murine leukemia (L1210)	18 μg/mL	[71]
Irimoteolide-12a	*Amphidinium* sp. (strain KCA09053)	Human B lymphocyte DG-75	18 μg/mL	[80]
Amphidinol 22	*Amphidinium carterae*	Human skin melanoma (A2058)	27 μg/mL	[62]
Amphidinol 22	*Amphidinium carterae*	Breast adenocarcinoma (MCF7)	27.5 μg/mL	[62]
Chloroform extract	*Amphidinium carterae*	Promyelotic leukemia (HL-60)	50 μg/mL	[84]

## Data Availability

No new data were created or analyzed in this study. Data sharing is not applicable to this article.
